# Association between type 2 diabetes status and prevalence of liver steatosis and fibrosis among adults aged ≥ 40 years

**DOI:** 10.1186/s12902-022-01046-y

**Published:** 2022-05-13

**Authors:** Jun Chen, Piao Hu, Yanfei Wang, Zhongxin Zhu

**Affiliations:** 1 Department of Endocrinology, The First People’s Hospital of Xiaoshan District, Xiaoshan Affiliated Hospital of Wenzhou Medical University, Hangzhou, 311200 Zhejiang China; 2 Department of Infectious Diseases, The First People’s Hospital of Xiaoshan District, Xiaoshan Affiliated Hospital of Wenzhou Medical University, Hangzhou, 311200 Zhejiang China; 3 Department of Medical Oncology, The First People’s Hospital of Xiaoshan District, Xiaoshan Affiliated Hospital of Wenzhou Medical University, Hangzhou, 311200 Zhejiang China; 4 Clinical Research Center, The First People’s Hospital of Xiaoshan District, Xiaoshan Affiliated Hospital of Wenzhou Medical University, Hangzhou, 311200 Zhejiang China

**Keywords:** Diabetes, Controlled attenuation parameter, Liver steatosis, Liver stiffness, Fibrosis

## Abstract

**Background:**

Type 2 diabetes mellitus (T2DM) and non-alcoholic fatty liver disease frequently coexist and share pathophysiological manifestations. This study aimed to explore the association between T2DM status and prevalence of liver steatosis and fibrosis, identified using the controlled attenuation parameter and liver stiffness measurement attained via liver ultrasound transient elastography.

**Methods:**

This was a cross-sectional analysis of data collected in the National Health and Nutrition Examination Survey for 2017–2018. Multivariable logistic regression model was used to evaluate the association between T2DM and prevalence of liver steatosis and fibrosis. Subgroup analyses, stratified by sex age, race, and body mass index (BMI), were further performed.

**Results:**

Of the 2,780 participants aged ≥ 40 years enrolled, 749 had T2DM, and 2,031 did not. After adjustment for potential confounders, T2DM was associated with a higher prevalence of liver steatosis (OR = 1.7, 95% CI, 1.3–2.1). This T2DM-related prevalence was higher among women (OR = 1.8, 95% CI, 1.3–2.5) and in the non-Hispanic Black (OR = 1.8, 95% CI, 1.1–3.0), other race (OR = 1.9, 95% CI, 1.2–3.0), and BMI < 25 kg/m^2^ (OR = 2.0, 95% CI, 1.1–3.8) groups. T2DM was also associated with a significantly higher prevalence of fibrosis (OR = 2.0, 95% CI, 1.5–2.7), with this association being more prominent for the other race (OR = 2.9, 95% CI, 1.5–5.5) and BMI < 25 kg/m^2^ (OR = 3.3, 95% CI: 1.3–8.8) groups.

**Conclusions:**

Our findings indicated a positive association between T2DM status and prevalence of hepatic steatosis and fibrosis. This association was more prominent for individuals with a BMI < 25 kg/m^2^ and was influenced by race-specific effects.

## Background

Non-alcoholic fatty liver disease (NAFLD) is the most common chronic liver disease and has become a major global health concern [1, 2]. In recent years, the prevalence of NAFLD has been rising progressively, along with type 2 diabetes mellitus (T2DM), which has reached epidemic levels [3]. T2DM is recognized as one of the strongest risk factors for the progression of NAFLD to non-alcoholic steatohepatitis, advanced fibrosis, or cirrhosis [4]. T2DM and NAFLD frequently coexist, with shared pathophysiological manifestations of excessive fat accumulation and insulin resistance [5].

The diagnosis of NAFLD is based on the detection of steatosis on liver biopsy and imaging techniques, after the exclusion of hepatic fatty infiltration and other causes of abnormal transaminase values via laboratory screening and medical history [6]. As a non-invasive imaging tool, liver ultrasound transient elastography (TE) provides excellent diagnostic accuracy for liver steatosis and advanced liver diseases in adults [7]. The latest cycle of the National Health and Nutrition Examination Survey (NHANES) includes liver ultrasound TE for the diagnosis of liver steatosis and advanced liver disease based on the controlled attenuation parameter (CAP) and liver stiffness measurement (LSM). Herein, we explored the association between T2DM status and prevalence of liver steatosis and fibrosis, indicated by the CAP and LSM, among adults aged ≥ 40 years using the NHANES database.

## Methods

### Study population

This cross-sectional study used data from the NHANES database (2017–2018 cycle). The NHANES is a program designed to provide objective health data of the population of the United States. The methodology and data collection for the NHANES are freely available (http://www.cdc.gov/nchs/nhanes.htm) and have been fully described [8]. Among 3,882 adults aged ≥ 40 years whose data were available in the database, the following were excluded: 441 for whom serum glucose or glycohemoglobin (HbA1c) data were unavailable; 234 without CAP or LSM data; 375 due to the presence of hepatitis B surface antigen, hepatitis C antibody, or a history of significant alcohol consumption (men: > 30 g/day; women: > 20 g/day) [9], 26 aged < 30 years at the time of diabetes mellitus (DM) onset; and 26 without body mass index (BMI) data. We included 2,780 participants in the final analysis.

The National Center for Health Statistics Research Ethics Review Board approved the survey protocol and all participants provided written informed consent for data collection and the use of their information for research.

Our study is compliant with the Guidelines for the STrengthening the Reporting of OBservational studies in Epidemiology (STROBE) guidelines [10].

### Study variables

The exposure for our study is the T2DM status, defined according to the following criteria: participants being informed that they had DM by their doctor, age at time of DM diagnosis ≥ 30 years; and/or a HbA1c level ≥ 6.5% [11]. Outcomes on liver ultrasound TE were measured using a FibroScan® system (model 502, V2 Touch) and included CAP, with a value ≥ 274 dB/m indicative of liver steatosis [12], and LSM, with a median value ≥ 8 kPa indicative of significant fibrosis [13], provided by the liver ultrasound TE on a FibroScan® model 502 V2 Touch equipped with a medium or extra large probe. The following demographic and clinical variables were also collected as covariates in our analyses: age; sex; race; level of education; ratio of family income to poverty; level of moderate recreational activities; history of smoking ≥ 100 cigarettes; BMI; and blood urea nitrogen (BUN) levels, total cholesterol, uric acid, gamma-glutamyl transpeptidase (GGT), aspartic acid transferase, alanine amino transferase (ALT), alkaline phosphatase (ALP), and serum glucose.

### Statistical analysis

All analyses were performed using statistical software R (version 3.4.3) and EmpowerStats (X&Y Solutions, Boston, MA), with a P-value < 0.05 considered significant. Multivariable logistic regression model was used to evaluate the association between T2DM status and prevalence of liver steatosis and fibrosis. Three statistical models were constructed: model 1, no adjustment for covariates; model 2, adjusted for age, sex, and race; and model 3, adjusted for all covariates presented in Table 1. Subgroup analyses, stratified by sex, age, race and, BMI were further performed.

**Table 1:** Characteristic of study sample with and without type 2 diabetes

	Non-diabetes (*n* = 2,031)	Type 2 diabetes (*n* = 749)	*P* value
Age (years)	59.5 ± 11.8	64.3 ± 10.4	< 0.001
Sex (%)			< 0.001
Men	45.6	53.5	
Women	54.4	46.5	
Race (%)			< 0.001
Non-Hispanic White	37.2	29.9	
Non-Hispanic Black	21.9	24.0	
Mexican American	11.9	16.0	
Other race	29.0	30.0	
Educational level (%)			< 0.001
Less than high school	19.9	27.0	
High school	24.0	22.6	
More than high school	56.0	50.5	
Body mass index (kg/m^2^)	29.3 ± 6.7	32.2 ± 7.3	< 0.001
Ratio of family income to poverty	2.7 ± 1.6	2.6 ± 1.6	0.231
Moderate recreational activities (%)			< 0.001
Yes	40.4	31.9	
No	59.6	68.1	
Smoked at least 100 cigarettes in life (%)			0.008
Yes	41.9	47.5	
No	58.1	52.5	
Glycohemoglobin (%)	5.6 ± 0.4	7.4 ± 1.5	< 0.001
Serum glucose (mmol/L)	5.3 ± 0.7	7.9 ± 3.5	< 0.001
Alkaline phosphatase (U/L)	80.7 ± 24.4	85.6 ± 30.9	< 0.001
Alanine amino transferase (IU/L)	20.9 ± 12.9	22.9 ± 15.8	< 0.001
Aspartic acid transferase (IU/L)	21.4 ± 9.0	21.8 ± 13.1	0.372
Gamma-glutamyl transpeptidase (IU/L)	30.0 ± 37.8	37.5 ± 44.0	< 0.001
Serum uric acid (umol/L)	323.5 ± 85.6	343.3 ± 94.7	< 0.001
Blood urea nitrogen (mmol/L)	5.6 ± 2.0	6.4 ± 3.0	< 0.001
Total cholesterol ((mmol/L)	5.1 ± 1.0	4.6 ± 1.2	< 0.001
Median controlled attenuation parameter (dB/m)	264.5 ± 58.2	301.8 ± 59.0	< 0.001
Liver steatosis (%)			< 0.001
Yes	43.8	67.6	
No	56.2	32.4	
Median liver stiffness (kpa)	5.7 ± 5.1	7.6 ± 6.5	< 0.001
Significant fibrosis (%)			< 0.001
Yes	9.4	25.4	
No	90.6	74.6	

Mean ± SD for continuous variables: *P* value was calculated by one-way ANOVA (normal distribution) and Kruskal–Wallis H (skewed distribution) test

% for categorical variables: *P* value was calculated by chi-square test

## Results

The characteristics of the study sample, according to T2DM status, are presented in Table 1. Of the 2,780 participants enrolled, 749 had a diagnosis of T2DM, with the other 2,031 classified in the non-DM group. Compared to the non-DM group, participants with T2DM were older, had a higher BMI and levels of ALP, ALT, GGT, uric acid, and BUN, had higher CAP and LSM values, a higher proportion of liver steatosis and significant fibrosis, and a lower level of total cholesterol.

### Association between T2DM status and CAP

After adjustment for potential confounding factors, T2DM status was positively associated with CAP (β = 16.8, 95% CI, 11.8–21.8; Table 2). On subgroup analyses, this positive association was more prominent among women (β = 19.7, 95% CI, 12.6–26.7) than it was among men (β = 12.2, 95% CI, 4.9–19.4), and in the non-hispanic black (β = 19.5, 95% CI, 9.1–29.9), other race (β = 19.4, 95% CI, 10.2–28.5), and BMI < 25 kg/m^2^ (β = 19.8, 95% CI, 8.7–31.0) groups.

**Table 2:** Association between type 2 diabetes status and controlled attenuation parameter (dB/m)

	Model 1 β (95% CI, P)	Model 2 β (95% CI, P)	Model 3 β (95% CI, P)
Non- diabetes	Reference	Reference	Reference
Type 2 diabetes	37.4 (32.5, 42.3) < 0.001	39.1 (34.2, 44.1) < 0.001	16.8 (11.8, 21.8) < 0.001
Stratified by sex			
Men (*n* = 1,328)			
Non- diabetes	Reference	Reference	Reference
Type 2 diabetes	31.3 (24.2, 38.5) < 0.001	34.0 (26.7, 41.2) < 0.001	12.2 (4.9, 19.4) 0.001
Women (*n* = 1,452)			
Non- diabetes	Reference	Reference	Reference
Type 2 diabetes	41.7 (35.0, 48.4) < 0.001	44.2 (37.4, 51.0) < 0.001	19.7 (12.6, 26.7) < 0.001
Stratified by age			
40–59 age group (*n* = 1,240)			
Non- diabetes	Reference	Reference	Reference
Type 2 diabetes	47.2 (38.6, 55.7) < 0.001	47.1 (38.6, 55.7) < 0.001	19.1 (10.4, 27.8) < 0.001
60–80 age group (*n* = 1,540)			
Non- diabetes	Reference	Reference	Reference
Type 2 diabetes	35.0 (29.0, 41.1) < 0.001	34.3 (28.3, 40.3) < 0.001	15.4 (9.0, 21.7) < 0.001
Stratified by race			
Non-Hispanic White (*n* = 979)			
Non- diabetes	Reference	Reference	Reference
Type 2 diabetes	41.6 (32.7, 50.5) < 0.001	43.5 (34.6, 52.4) < 0.001	13.2 (3.9, 22.5) 0.005
Non-Hispanic Black (*n* = 624)			
Non- diabetes	Reference	Reference	Reference
Type 2 diabetes	34.6 (24.5, 44.7) < 0.001	37.5 (27.4, 47.6) < 0.001	19.5 (9.1, 29.9) < 0.001
Mexican American (*n* = 362)			
Non- diabetes	Reference	Reference	Reference
Type 2 diabetes	29.9 (17.6, 42.2) < 0.001	29.9 (16.9, 42.8) < 0.001	12.0 (-1.1, 25.2) 0.074
Other race (*n* = 815)			
Non- diabetes	Reference	Reference	Reference
Type 2 diabetes	39.1 (30.5, 47.8) < 0.001	38.0 (29.1, 47.0) < 0.001	19.4 (10.2, 28.5) < 0.001
Stratified by body mass index (BMI)			
BMI < 25 (kg/m^2^) (*n* = 632)			
Non- diabetes	Reference	Reference	Reference
Type 2 diabetes	32.4 (22.2, 42.6) < 0.001	29.3 (19.0, 39.5) < 0.001	19.8 (8.7, 31.0) < 0.001
BMI ≥ 25, < 30 (kg/m^2^) (*n* = 951)			
Non- diabetes	Reference	Reference	Reference
Type 2 diabetes	27.5 (19.7, 35.4) < 0.001	24.3 (16.2, 32.4) < 0.001	14.4 (5.0, 23.8) 0.003
BMI ≥ 30 (kg/m^2^) (*n* = 1,197)			
Non- diabetes	Reference	Reference	Reference
Type 2 diabetes	25.0 (18.5, 31.6) < 0.001	27.2 (20.7, 33.7) < 0.001	15.9 (8.7, 23.0) < 0.001

Model 1: no covariates were adjusted

Model 2: age, sex, race were adjusted

Model 3: age, sex, race, educational level, body mass index, ratio of family income to poverty, moderate recreational activities, smoked at least 100 cigarettes in life, blood urea nitrogen, total cholesterol, serum uric acid, alkaline phosphatase, alanine amino transferase, aspartic acid transferase, Gamma-glutamyl transpeptidase, and serum glucose were adjusted

### Association between T2DM status and risk of liver steatosis

In the fully adjusted model (Table 3), T2DM status was positively associated with prevalence of liver steatosis (OR = 1.7, 95% CI, 1.3–2.1). On subgroup analyses, this positive association was more prominent among women (OR = 1.8, 95% CI, 1.3–2.5) than men (OR = 1.5, 95% CI: 1.0–2.1), and in the non-Hispanic Black (OR = 1.8, 95% CI, 1.1–3.0), other race (OR = 1.9, 95% CI, 1.2–3.0), and BMI < 25 kg/m^2^ (OR = 2.0, 95% CI, 1.1–3.8) groups.

**Table 3:** Association between type 2 diabetes status and prevalence of liver steatosis

	Model 1 OR (95% CI, P)	Model 2 OR (95% CI, P)	Model 3 OR (95% CI, P)
Non- diabetes	Reference	Reference	Reference
Type 2 diabetes	2.7 (2.2, 3.2) < 0.001	2.9 (2.4, 3.4) < 0.001	1.7 (1.3, 2.1) < 0.001
Stratified by sex			
Men (*n* = 1,328)			
Non- diabetes	Reference	Reference	Reference
Type 2 diabetes	2.3 (1.8, 3.0) < 0.001	2.5 (2.0, 3.3) < 0.001	1.5 (1.0, 2.1) 0.033
Women (*n* = 1,452)			
Non- diabetes	Reference	Reference	Reference
Type 2 diabetes	3.0 (2.3, 3.9) < 0.001	3.2 (2.5, 4.1) < 0.001	1.8 (1.3, 2.5) 0.001
Stratified by age			
40–59 age group (*n* = 1,240)			
Non- diabetes	Reference	Reference	Reference
Type 2 diabetes	3.4 (2.4, 4.7) < 0.001	3.4 (2.5, 4.8) < 0.001	1.4 (0.9, 2.2) 0.190
60–80 age group (*n* = 1,540)			
Non- diabetes	Reference	Reference	Reference
Type 2 diabetes	2.6 (2.1, 3.2) < 0.001	2.6 (2.1, 3.3) < 0.001	1.8 (1.3, 2.4) < 0.001
Stratified by race			
Non-Hispanic White (*n* = 979)			
Non- diabetes	Reference	Reference	Reference
Type 2 diabetes	2.9 (2.1, 4.0) < 0.001	3.0 (2.2, 4.2) < 0.001	1.2 (0.8, 1.9) 0.414
Non-Hispanic Black (*n* = 624)			
Non- diabetes	Reference	Reference	Reference
Type 2 diabetes	2.4 (1.7, 3.4) < 0.001	2.6 (1.8, 3.8) < 0.001	1.8 (1.1, 3.0) 0.014
Mexican American (n = 362)			
Non- diabetes	Reference	Reference	Reference
Type 2 diabetes	2.6 (1.6, 4.2) < 0.001	2.6 (1.5, 4.3) < 0.001	1.7 (0.9, 3.4) 0.129
Other race (*n* = 815)			
Non- diabetes	Reference	Reference	Reference
Type 2 diabetes	2.9 (2.1, 4.0) < 0.001	2.9 (2.1, 4.1) < 0.001	1.9 (1.2, 3.0) 0.003
Stratified by body mass index (BMI)			
BMI < 25 (kg/m^2^) (*n* = 632)			
Non- diabetes	Reference	Reference	Reference
Type 2 diabetes	3.0 (1.9, 4.8) < 0.001	2.6 (1.6, 4.3) < 0.001	2.0 (1.1, 3.8) 0.023
BMI ≥ 25, < 30 (kg/m^2^) (*n* = 951)			
Non- diabetes	Reference	Reference	Reference
Type 2 diabetes	2.2 (1.6, 2.9) < 0.001	2.0 (1.5, 2.8) < 0.001	1.5 (1.0, 2.2) 0.074
BMI ≥ 30 (kg/m^2^) (*n* = 1,197)			
Non- diabetes	Reference	Reference	Reference
Type 2 diabetes	2.0 (1.5, 2.6) < 0.001	2.1 (1.6, 2.9) < 0.001	1.6 (1.1, 2.2) 0.012

Model 1: no covariates were adjusted

Model 2: age, sex, race were adjusted

Model 3: age, sex, race, educational level, body mass index, ratio of family income to poverty, moderate recreational activities, smoked at least 100 cigarettes in life, blood urea nitrogen, total cholesterol, serum uric acid, alkaline phosphatase, alanine amino transferase, aspartic acid transferase, Gamma-glutamyl transpeptidase, and serum glucose were adjusted

### Association between T2DM status and LSM

In the fully adjusted model, there was a positive association between T2DM status and LSM (β = 0.8, 95% CI, 0.2–1.3; Table 4). On subgroup analyses, this positive association was only identified among men (β = 0.9, 95% CI, 0.0–1.8) and in the 40–59 age (β = 1.0, 95% CI, 0.1–1.8), other race (β = 1.8, 95% CI, 0.8–2.9), and BMI ≥ 30 kg/m^2^ (β = 1.0, 95% CI, 0.1–1.9) groups.

**Table 4:** Association between type 2 diabetes status and liver stiffness (kpa)

	Model 1 β (95% CI, P)	Model 2 β (95% CI, P)	Model 3 β (95% CI, P)
Non- diabetes	Reference	Reference	Reference
Type 2 diabetes	1.9 (1.4, 2.3) < 0.001	1.8 (1.4, 2.3) < 0.001	0.8 (0.2, 1.3) 0.006
Stratified by sex			
Men (*n* = 1,328)			
Non- diabetes	Reference	Reference	Reference
Type 2 diabetes	1.9 (1.2, 2.7) < 0.001	2.0 (1.2, 2.8) < 0.001	0.9 (0.0, 1.8) 0.046
Women (*n* = 1,452)			
Non- diabetes	Reference	Reference	Reference
Type 2 diabetes	1.7 (1.2, 2.3) < 0.001	1.7 (1.1, 2.2) < 0.001	0.4 (-0.2, 1.1) 0.173
Stratified by age			
40–59 age group (*n* = 1,240)			
Non- diabetes	Reference	Reference	Reference
Type 2 diabetes	2.6 (1.9, 3.3) < 0.001	2.5 (1.8, 3.3) < 0.001	1.0 (0.1, 1.8) 0.027
60–80 age group (*n* = 1,540)			
Non- diabetes	Reference	Reference	Reference
Type 2 diabetes	1.5 (0.9, 2.1) < 0.001	1.5 (0.9, 2.1) < 0.001	0.7 (-0.0, 1.4) 0.058
Stratified by race			
Non-Hispanic White (*n* = 979)			
Non- diabetes	Reference	Reference	Reference
Type 2 diabetes	2.2 (1.3, 3.0) < 0.001	2.1 (1.3, 2.9) < 0.001	0.2 (-0.7, 1.2) 0.631
Non-Hispanic Black (*n* = 624)			
Non- diabetes	Reference	Reference	Reference
Type 2 diabetes	0.7 (-0.3, 1.8) 0.170	0.7 (-0.3, 1.8) 0.176	0.0 (-1.2, 1.2) 0.980
Mexican American (*n *= 362)			
Non- diabetes	Reference	Reference	Reference
Type 2 diabetes	2.1 (1.3, 2.8) < 0.001	1.8 (1.0, 2.7) < 0.001	0.7 (-0.2, 1.6) 0.108
Other race (*n* = 815)			
Non- diabetes	Reference	Reference	Reference
Type 2 diabetes	2.5 (1.6, 3.4) < 0.001	2.4 (1.5, 3.3) < 0.001	1.8 (0.8, 2.9) < 0.001
Stratified by body mass index (BMI)			
BMI < 25 (kg/m^2^) (*n* = 632)			
Non- diabetes	Reference	Reference	Reference
Type 2 diabetes	0.9 (0.3, 1.5) 0.003	0.9 (0.2, 1.5) 0.006	0.5 (-0.2, 1.2) 0.130
BMI ≥ 25, < 30 (kg/m^2^) (*n* = 951)			
Non- diabetes	Reference	Reference	Reference
Type 2 diabetes	1.1 (0.4, 1.9) 0.004	0.7 (-0.1, 1.5) 0.076	0.6 (-0.4, 1.6) 0.226
BMI ≥ 30 (kg/m^2^) (*n* = 1,197)			
Non- diabetes	Reference	Reference	Reference
Type 2 diabetes	2.0 (1.2, 2.8) < 0.001	2.0 (1.2, 2.8) < 0.001	1.0 (0.1, 1.9) 0.032

Model 1: no covariates were adjusted

Model 2: age, sex, race were adjusted

Model 3: age, sex, race, educational level, body mass index, ratio of family income to poverty, moderate recreational activities, smoked at least 100 cigarettes in life, blood urea nitrogen, total cholesterol, serum uric acid, alkaline phosphatase, alanine amino transferase, aspartic acid transferase, Gamma-glutamyl transpeptidase, and serum glucose were adjusted

### Association between T2DM status and risk of significant fibrosis

In the fully adjusted model, T2DM status and prevalence of significant fibrosis were positively correlated (OR = 2.0, 95% CI, 1.5–2.7) (Table 5). On subgroup analyses, this positive association was more prominent among individuals in the other race (OR = 2.9, 95% CI, 1.5–5.5) and BMI < 25 kg/m^2^ (OR = 3.3, 95% CI, 1.3–8.8) groups.

**Table 5:** Association between type 2 diabetes status and prevalence of significant fibrosis

	Model 1 OR (95% CI, P)	Model 2 OR (95% CI, P)	Model 3 OR (95% CI, P)
Non- diabetes	Reference	Reference	Reference
Type 2 diabetes	3.3 (2.6, 4.1) < 0.001	3.3 (2.6, 4.2) < 0.001	2.0 (1.5, 2.7) < 0.001
Stratified by sex			
Men (*n* = 1,328)			
Non- diabetes	Reference	Reference	Reference
Type 2 diabetes	2.9 (2.2, 4.0) < 0.001	3.1 (2.3, 4.3) < 0.001	1.8 (1.2, 2.8) 0.004
Women (*n* = 1,452)			
Non- diabetes	Reference	Reference	Reference
Type 2 diabetes	3.6 (2.6, 5.0) < 0.001	3.6 (2.5, 5.0) < 0.001	2.0 (1.3, 3.1) 0.003
Stratified by age			
40–59 age group (*n* = 1,240)			
Non- diabetes	Reference	Reference	Reference
Type 2 diabetes	4.6 (3.2, 6.6) < 0.001	4.5 (3.1, 6.5) < 0.001	2.3 (1.4, 3.9) 0.002
60–80 age group (*n* = 1,540)			
Non- diabetes	Reference	Reference	Reference
Type 2 diabetes	2.7 (2.1, 3.7) < 0.001	2.7 (2.0, 3.7) < 0.001	2.0 (1.4, 2.9) < 0.001
Stratified by race			
Non-Hispanic White (*n* = 979)			
Non- diabetes	Reference	Reference	Reference
Type 2 diabetes	3.5 (2.4, 5.2) < 0.001	3.5 (2.4, 5.3) < 0.001	2.0 (1.2, 3.4) 0.011
Non-Hispanic Black (*n* = 624)			
Non- diabetes	Reference	Reference	Reference
Type 2 diabetes	1.9 (1.2, 2.9) 0.008	1.9 (1.2, 3.0) 0.006	1.7 (1.0, 3.1) 0.067
Mexican American (*n* = 362)			
Non- diabetes	Reference	Reference	Reference
Type 2 diabetes	3.0 (1.7, 5.3) < 0.001	3.0 (1.6, 5.5) < 0.001	1.6 (0.7, 3.7) 0.228
Other race (*n* = 815)			
Non- diabetes	Reference	Reference	Reference
Type 2 diabetes	5.5 (3.5, 8.6) < 0.001	5.5 (3.4, 8.8) < 0.001	2.9 (1.5, 5.5) 0.001
Stratified by body mass index (BMI)			
BMI < 25 (kg/m^2^) (*n* = 632)			
Non- diabetes	Reference	Reference	Reference
Type 2 diabetes	2.4 (1.2, 4.7) 0.013	2.3 (1.1, 4.8) 0.021	3.3 (1.3, 8.8) 0.015
BMI ≥ 25, < 30 (kg/m^2^) (*n* = 951)			
Non- diabetes	Reference	Reference	Reference
Type 2 diabetes	2.7 (1.6, 4.4) < 0.001	2.2 (1.3, 3.8) 0.003	1.5 (0.7, 3.1) 0.257
BMI ≥ 30 (kg/m^2^) (*n* = 1,197)			
Non- diabetes	Reference	Reference	Reference
Type 2 diabetes	2.9 (2.2, 3.8) < 0.001	2.9 (2.2, 3.9) < 0.001	2.3 (1.6, 3.3) < 0.001

Model 1: no covariates were adjusted

Model 2: age, sex, race were adjusted

Model 3: age, sex, race, educational level, body mass index, ratio of family income to poverty, moderate recreational activities, smoked at least 100 cigarettes in life, blood urea nitrogen, total cholesterol, serum uric acid, alkaline phosphatase, alanine amino transferase, aspartic acid transferase, Gamma-glutamyl transpeptidase, and serum glucose were adjusted

## Discussion

In this study, we evaluated the association between T2DM status and prevalence of liver steatosis and fibrosis among adults aged ≥ 40 years, and found that T2DM was associated with a significantly higher prevalence of liver steatosis, with this association being more prominent among women and the non-Hispanic Black, other race, and BMI < 25 kg/m^2^ groups. T2DM also positively correlated with the prevalence of significant fibrosis, which was more prominent in the other race and BMI < 25 kg/m^2^ groups.

The bidirectional and mutual relationship between T2DM and NAFLD has been highlighted by epidemiological studies, with NAFLD increasing the risk of T2DM incidence, and T2DM increasing the risk of NAFLD incidence and progression [14]. A recent meta-analysis showed that the pooled prevalence of NAFLD among adults with T2DM was around 60%, with this prevalence varying by age and by BMI [15]. Compared to non-diabetes patients, those with combined NAFLD and T2DM have a higher risk of NAFLD progression [16]. A previous NHANES study (NHANES III) revealed that diabetes was associated with all-cause and cardiovascular mortality among individuals with NAFLD [17].

Among the non-invasive tests for NAFLD, TE is the most widely used for the assessment of liver fibrosis [18]. A higher prevalence of advanced fibrosis assessed via TE was observed among patients with T2DM [19–22]. The results of a recent NHANES study reported high rates of hepatic steatosis and fibrosis, diagnosed by CAP and LSM, among patients with T2DM, but with race-dependent differences [23]. Similarly, in our study, the association between T2DM status and CAP or LSM was prominent in some races, but not in others, including a non-significant association among Mexican–American individuals.

The common pathophysiological mechanisms shared by NAFLD and T2DM include a series of metabolic changes; in particular, changes in the white adipose tissue may play a central role in the initiation of both NAFLD and T2DM [24]. In 2020, an international panel of experts from 22 countries proposed the novel term “metabolic dysfunction-associated fatty liver disease” to replace NAFLD, which further emphasizes the strong association between T2DM and NAFLD [25]. NAFLD and T2DM not only have almost the same risk factors, but also have synergistic effects on each other’s disease progression and complications. Therefore, routine screening for T2DM among individuals with NAFLD and lifestyle changes, including diet modifications and physical activity, are recommended for the prevention and management of both T2DM and NAFLD.

Our study had some limitations. First, as this was a cross-sectional study, no causality could be established. Second, we excluded participants with age of DM onset of < 30 years of age to minimize the number of participants with T1DM, as previously described [26, 27], as the NHANES database does not differentiate diabetes by type. Third, the values of CAP defining hepatic steatosis and LSM defining significant fibrosis are both inconsistent among different studies using NAHENS 2017–2018 database [13, 28, 29]. Thus, the sensitivity and specificity of TE test may vary depending on the cut-off values. Fourth, differences in measurements depending on the probe used in FibroScan have been demonstrated in previous reports [30, 31]. However, the elastography exams were performed by trained and certified technicians, according to the manufacturer guidelines [32]. Last, self-reported confounders may be susceptible to individual biases. This source of bias was minimized by the utilization of the NHANES data, which is collected by trained personnel through established procedures.

## Conclusion

In conclusion, our findings indicate that T2DM is positively associated with prevalence of hepatic steatosis and fibrosis. This association was more prominent for individuals with a BMI < 25 kg/m^2^ and was influenced by race-specific effects. Routine screening for T2DM among individuals with NAFLD may contribute to the prevention and the management of both T2DM and NAFLD.

## Data Availability

The datasets generated and analysed during the current study are available in the NHANES website (http://www.cdc.gov/nchs/nhanes.htm).

## Abbreviations

NAFLD: Non-alcoholic fatty liver disease
T2DM: Type 2 diabetes mellitus
TE: Transient elastography
NHANES: National Health and Nutrition Examination Survey
CAP: Controlled attenuation parameter
LSM: Liver stiffness measurement
HbA1c: Glycohemoglobin
BMI: Body mass index
DM: Diabetes mellitus
BUN: Blood urea nitrogen
GGT: Gamma-glutamyl transpeptidase
ALT: Alanine amino transferase
ALP: Alkaline phosphatase
